# Pyrethroid susceptibility of malaria vectors in four Districts of western Kenya

**DOI:** 10.1186/1756-3305-7-310

**Published:** 2014-07-04

**Authors:** Eric Ochomo, Nabie M Bayoh, Luna Kamau, Francis Atieli, John Vulule, Collins Ouma, Maurice Ombok, Kiambo Njagi, David Soti, Evan Mathenge, Lawrence Muthami, Teresa Kinyari, Krishanthi Subramaniam, Immo Kleinschmidt, Martin James Donnelly, Charles Mbogo

**Affiliations:** 1 School of Public Health and Community Development, Maseno University, Maseno, Kenya; 2 KEMRI/CDC Research and Public Health Collaboration, PO Box 1578, Kisumu 40100, Kenya; 3 KEMRI –Centre for Biotechnology and Research Development, Nairobi, Kenya; 4 KEMRI- Centre for Global Health Research, Kenya Medical Research Institute, PO Box 1578 Kisumu 40100, Kenya; 5 Division of Malaria Control (DOMC), Ministry of Health, Nairobi, Kenya; 6 KEMRI-Eastern and Southern Africa Centre of International Parasite Control, Nairobi, Kenya; 7 KEMRI- Centre for Public Health Research, Nairobi, Kenya; 8 Department of Medical Physiology, University of Nairobi, Nairobi, Kenya; 9 Department of Vector Biology, Liverpool School of Tropical Medicine, Liverpool, UK; 10 Department of Infectious Disease Epidemiology, London School of Hygiene and Tropical Medicine, London, UK; 11 Kenya Medical Research Institute, Centre for Geographic Medicine Research-Coast, Kilifi, Kenya; 12 Malaria Public Health Department, KEMRI-Wellcome Trust Research Program, Nairobi, Kenya

**Keywords:** Mortality, Insecticide resistance, Pyrethroids, IRS

## Abstract

**Background:**

Increasing pyrethroid resistance in malaria vectors has been reported in western Kenya where long lasting insecticidal nets (LLINs) and indoor residual spraying (IRS) are the mainstays of vector control. To ensure the sustainability of insecticide-based malaria vector control, monitoring programs need to be implemented. This study was designed to investigate the extent and distribution of pyrethroid resistance in 4 Districts of western Kenya (Nyando, Rachuonyo, Bondo and Teso). All four Districts have received LLINs while Nyando and Rachuonyo Districts have had IRS campaigns for 3–5 years using pyrethroids. This study is part of a programme aimed at determining the impact of insecticide resistance on malaria epidemiology.

**Methods:**

Three day old adult mosquitoes from larval samples collected in the field, were used for bioassays using the WHO tube bioassay, and mortality recorded 24 hours post exposure. Resistance level was assigned based on the 2013 WHO guidelines where populations with <90% mortality were considered resistant. Once exposed, samples were identified to species using PCR.

**Results:**

*An. arabiensis* comprised at least 94% of all *An. gambiae* s.l. in Bondo, Rachuonyo and Nyando. Teso was a marked contrast case with 77% of all samples being *An. gambiae* s.s. Mortality to insecticides varied widely between clusters even in one District with mortality to deltamethrin ranging from 45-100%, while to permethrin the range was 30-100%. Mortality to deltamethrin in Teso District was < 90% in 4 of 6 clusters tested in *An arabiensis* and <90% in *An. gambiae* s.s in 5 of 6 clusters tested. To permethrin, mortality ranged between 5.9-95%, with <90% mortality in 9 of 13 and 8 of 13 in *An. arabiensis* and *An. gambiae* s.s. respectively. Cluster specific mortality of *An. arabiensis* between permethin and deltamethrin were not correlated (*Z* = 2.9505, *P* = 0.2483).

**Conclusion:**

High levels of pyrethroid resistance were observed in western Kenya. This resistance does not seem to be associated with either species or location. Insecticide resistance can vary within small geographical areas and such heterogeneity may make it possible to evaluate the impact of resistance on malaria and mosquito parameters within similar eco-epidemiological zones.

## Background

Vector control remains central to the fight against malaria, with ITNs being a central component of the WHO global strategy [1]. The use of ITNs has been reported to contribute to a reduction in various entomologic [2,3] and epidemiologic indices [4-6] and, most importantly a reduction in morbidity and mortality in adults and children under the age of five [7-9]. Thus, ITN use has been a major contributor to the declines in malaria endemicity in Africa. The recent WHO position statement on IRS has brought an important change in the landscape of malaria control in Africa. The use of IRS has increased almost 6 fold since 2001 [10] and has stimulated a renewed interest in malaria prevention with an emphasis on vector control with success seen in several parts of sub-Saharan Africa [6,11]. However, there has been an upsurge of insecticide resistance in different parts of the world [12-17] resulting in fears that the effectiveness of these control measures may be compromised. Data from different parts of Africa seem to suggest that insecticide resistance is as a result of selection pressure brought about by the use of insecticides in agriculture; for example in West African populations of *Anopheles gambiae* from the Cote d’ Ivoire and Burkina Faso resistance is thought to have been selected for by pyrethroids used in cotton farming [18-21]. In western Kenya, a reduction in susceptibility to pyrethroid insecticides was reported after one year of a large-scale permethrin impregnated bednet programme [5,22] and has since been reported in multiple sites [23,24].

Data from the Kenya Medical Research Institute/Centers for Disease Control and Prevention (KEMRI/CDC) Health demographic surveillance system (HDSS) suggest that malaria prevalence in western Kenya has declined from 60% in 2003 to 26% in 2008 followed by a rise to 40% in 2009. This was speculated to be possibly due to stock-outs of essential antimalarial drugs during a time of increased malaria transmission and disruption of services during civil unrest [25]. Although a different study speculated that reduced efficacy of ITNs, insecticide resistance in local vector populations and lack of proper ITN use may have contributed to the reduced malaria prevalence [26].

The Kenyan National Malaria Strategy has the vision of making Kenya malaria free by 2017 [27,28]. The current Kenyan national strategy for malaria control involves prompt diagnosis and treatment of suspected cases of uncomplicated malaria and vector control. The National Malaria Control Program advocates the use of ITNs in malaria endemic areas and IRS in endemic and epidemic prone areas. The insecticides of choice in both strategies are synthetic pyrethroids. This study is BMGF-funded and coordinated by the WHO and is part of a multinational programme currently ongoing in 5 countries; Benin, Cameroon, Sudan, India and Kenya to assess the impact of insecticide resistance in malaria vectors on the effectiveness of vector control interventions in a range of transmission settings. This article details the results of an insecticide resistance survey carried out in 80 clusters in 4 Districts in Kenya. The main objective of the survey was to determine the extent and distribution of pyrethroid resistance in local malaria vectors in western Kenya.

## Methods

### Study sites

**Figure 1:**
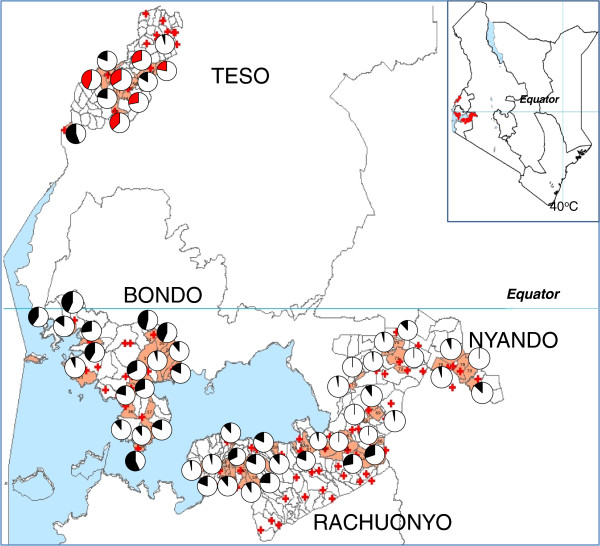
**Map of Kenya showing the study Districts (right) and map of the study Districts with clusters highlight in orange.** The red crosses represent health facilities. The pie charts indicate susceptibility status of mosquito populations to deltamethrin in the study clusters. The black charts indicating the resistance status of *An. arabiensis* while the red charts indicate resistance status of *An. gambiae* s.s.

**Figure 2:**
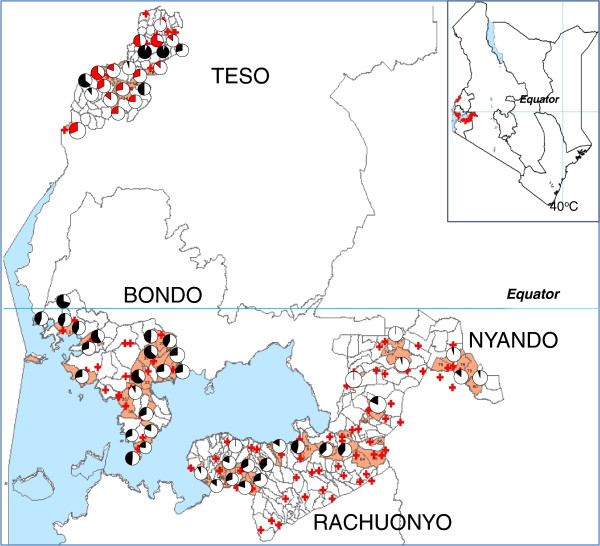
**The pie charts indicate susceptibility status of mosquito populations to permethrin in the study clusters.** The red charts indicate the resistance status of *An. Arabiensis,* while the black charts indicate resistance status of *An. gambiae* s.s.

This study was conducted in 4 malaria endemic Districts (Bondo, Rachuonyo, Nyando and Teso) in western Kenya. There were 2 distinct vector control interventions implemented in the Districts: Rachuonyo and Nyando: IRS combined with ITNs; Bondo and Teso: ITNs only (Figures 1 and 2). In each District, 20 sub-locations (clusters) were randomly selected for baseline resistance determination. In Kenya, sub-locations are the smallest administrative units and are composed of multiple households. Each sub-location can have as many as 10–30 villages each having about 100 households. Sample collections were conducted between July and September 2011.

Bondo District is located in Siaya County. The District has a population of 309,190 [29] and has recently been sub-divided into, Bondo and Rarieda Districts. For this study, Bondo refers to the larger, undivided District. The District borders Siaya to the North, Gem District to the East and Lake Victoria to the South. The altitude of the District rises from 1,140 m in the eastern parts to 1,400 m above sea level in the west. The Nzoia and Yala rivers traverse the District and enter Lake Victoria through the Yala Swamp. The District experiences bimodal rainfall; the relief and altitude influencing its distribution and amount. The District is drier in the western part towards Bondo District and is wetter towards the higher altitudes in the eastern part. In the highlands, the rainfall ranges between 800 mm and 2000 mm. The lower areas receive rainfall ranging from 800 and 1600 mm. The short rains occur between August and November. The mean minimum and maximum temperatures are 15°C and 30°C, respectively. Humidity is relatively high with mean evaporation being between 1800 mm to 2000 mm per annum. Malaria is a major problem in this District with a prevalence (children under 5 years) of about 30%. Bondo District has had long term use of bednets with net distribution having started in the early 2000s in some parts of the District. The early intervention with the bednets is associated with a drastic decline in *An. funestus* s.l. and a shift from a largely *An. gambiae* s.s. population to a largely *An. arabiensis* population [30], however, *An. funestus* s.l. have recently re-emerged in the District [31].

Rachuonyo District is in Homa Bay County and has a total population of 400,802 [29]. The District has recently been split into two, North and South Rachuonyo Districts. This study was conducted only in North Rachuonyo District. The District borders Lake Victoria to the North, Nyando District to the North East, Kisii District to the South and Homabay District to the West. The altitude of the District is around 1300 m above sea level with an annual rainfall around 1700 mm. The temperature ranges between 15 and 30°C. This District is also endemic for malaria with a prevalence of 26% in children under 5 years and has had rollout of bednets for over a decade and IRS from 2007 with coverage of >80% of the households for ITNs and >90% for IRS. Little has been published on the malaria vectors within this District but *An. arabiensis* and *An. funestus* are the most abundant (Bayoh *et al.* unpublished).

Nyando District is in Kisumu county and has a population of 389,351 [29]. The District is located adjacent to the Gulf of Winam on the northeast shore Lake Victoria. It is bordered to the North by Nandi District, to the east by Kericho District, to the South by Rachuonyo and Kisumu District to the West. There is a marked variation in altitude with the areas along the Nyando plateau having an altitude as low as 1100 m above sea level and is very prone to flooding, with other areas as high as 1540 m above sea level. Rice farming is a major economic activity and provides habitats conducive for the breeding of *An. arabiensis* and *An. funestus* mosquitoes, which drive transmission. This District has had long term intervention with bednets, the last mass campaign being in 2011 and more recently IRS in the same year. Malaria prevalence in this District is about 20% while bednet coverage defined as one bednet per two people is more than 85%.

Teso District is in Busia County and has a population of 252,884 [29]. The District has been split into Teso North and Teso South Districts though the study was conducted in both. It borders Busia District to the South West, Bumula District to the East, and Tororo District in Uganda to the North. The average altitude of the District is 1208 m above sea level. Trade is a major economic activity alongside subsistence farming. The prevalence of malaria in children under 5 years in this District is 32%. Bednets have been distributed in this District with the aim of universal coverage (2 people per bednet) and as such are the main intervention against malaria vectors. *An. gambiae* s.s is the major vector of malaria but *An. arabiensis* and *An. funestus* s.l. are also present but in lower proportions.

### Sample collection

#### ***Larval sampling and rearing***

*Anopheles* larvae were sampled from small, temporary and open habitats including ponds, potholes, tire tracks, rice fields, drainage channels and mud paths using the standard dipping method. Individual larvae were picked from the dippers using wide-mouthed plastic pipettes and placed in plastic tins for transportation to the KEMRI Centre for Global Health Research laboratories for rearing.

In the laboratory, the larvae were poured into larger trays and debris removed from the water. Larval samples from each cluster were pooled together and sorted according to instar stage and morphology. Similar instar stages were transferred to the same larval tray with all the trays being labelled with collection date and site. Larvae were reared on a mixture of fish food and brewer’s yeast provided daily and separation of instar stages was continued every 2 days. Upon pupation, pupae were collected and placed in pupa cups inside a labelled cage for emergence. Each day emerged adults were removed and placed in a new cage with sample identity and date of emergence. These were provided with 5% sugar solution as they awaited assay [24].

#### ***Bioassays***

Three-day-old adult *Anopheles* mosquitoes that had emerged from the larvae were exposed to insecticides using WHO impregnated papers for 1 h at standard concentrations using the WHO tube bioassay [32]. The mosquitoes were then maintained at 25 ± 2°C and 80 ± 5% RH and mortality was scored at 24 hours post-exposure using the new WHO guidelines [33]. Mosquitoes were exposed to permethrin (0.75%) and deltamethrin (0.05%) and mortality scored according to WHO 2013 guidelines. Only female *Anopheles* mosquitoes were included in the assays and analyses. Once mortality was scored, live mosquitoes were knocked down by freezing then all mosquitoes placed in individual tubes and frozen at -20°C for molecular analysis.

#### ***Species identification***

All of the samples exposed above were identified to species. Whole samples were used for DNA extraction using an ethanol precipitation method [34]. Polymerase chain reaction (PCR) was used to distinguish between the two sibling species of the *An. gambiae* sl*.* species complex, which have previously been observed in western Kenya, *An. gambiae* s.s*.* (molecular S-form) and *An. arabiensis*[35].

#### ***Data analysis***

Bioassay data was scored according to the guidelines by WHO, where populations with mortality >98% were regarded as susceptible, populations with 90 - 98% mortality were suspected to be resistant pending further tests while populations with <90% mortality were considered resistant. Chi-square analysis was used to compare mortalities between *An. gambiae* s.s. and *An. arabiensis* from clusters in Teso and Wilcoxon signed-rank test used to check for consistently higher mortalities in any one species. Lastly, the Kendall Tau test was used to test for the correlation between mortalities to permethrin and deltamethrin. All confidence intervals were calculated using the VassarStats confidence interval calculator (http://vassarstats.net/) [36,37].

## Results

### Species distribution

Apart from Teso, the most abundant vector species in all the Districts was *An. arabiensis*. This species comprised at least 94% of all *An. gambiae* s.l. in Bondo, Rachuonyo and Nyando. Teso was a marked contrast case with 77% of all samples being *An. gambiae* s.s. (Table 1).

**Table 1:** **Total number of *Anopheles gambiae *** **s.l. collected in the four study Districts**

**District.id**	**District**	**Total no of *Anopheles gambiae s.l.***	**Total no of *An. arabiensis***	**Total no of *An. gambiae s.s.***	**Proportions of *An. arabiensis***	**95% CIs around proportion of *Anopheles arabiensis***
1	Bondo	3372	3159	213	93.68	92.8-94.5
2	Rachuonyo	1487	1451	36	97.58	96.7-98.3
3	Teso	1332	306	1026	22.97	20.8-25.3
4	Nyando	1147	1101	46	95.99	94.7-97.0

A breakdown of the species composition for each District. The proportions of *An. arabiensis* and the 95% Confidence intervals around the proportions are also shown.

### Phenotypic assays

Mortality to insecticides varied widely between clusters even in one District with mortality to deltamethin ranging from 45 to 100%, while permethrin ranged from 30 to 100% (Figures 1 and 2). The datasets used in generating the pie charts are in Additional files 1 and 2. When tested against permethrin, mosquito populations from 19 of 20 clusters in Bondo, 2 of 7 clusters in Nyando, 11 of 13 clusters in Rachuonyo and 14 of 15 clusters in Teso had mortalities <90%. Against deltamethrin, 16 of 18 clusters in Bondo, 10 of 14 clusters in Rachuonyo, 5 of 18 clusters in Nyando and 6 of 6 clusters in Teso we had mortalities <90% which is the WHO threshold for resistance [33]. Susceptibility data for Bondo, Rachuonyo and Nyando could not be split down to the two species since *An. gambiae* s.s population was less than 6% in all the clusters in these sites. Overall, Teso had a higher proportion of *An. gambiae s.s*. even though the species distribution was not the same in all clusters. In two clusters in South Teso: Akiriamasi and Akiriamasit, more *An. arabiensis* were observed compared to *An gambiae* s.s. with higher resistance observed in *An gambiae* s.s. (χ^2^ = 7.89, P = 0.005; χ^2^ = 0.1, P = 0.75 respectively) with the rest of the clusters having more *An. gambiae* s.s. In Teso District, lower susceptibility to permethrin was observedin *An. arabiensis* compared to *An. gambiae* s.s. in Odioi and Kaliwa (Table 2). However, a Wilcoxon signed-rank test failed to show consistently higer/lower resistance in any one vector compared to the other (*Z* = 0.1, *P* = 0.9203). Correlation analysis using the Kendall Tau test did not show any correlation in cluster specific mortality of *An. arabiensis* between permethin and deltamethrin (*Z* = 2.9505, *P* = 0.2483).

**Table 2:** **Results of χ**^**2 **^**analysis of the differences in the phenotypic resistance of *An. gambiae *** **s.s. versus *An. arabiensis *** **when exposed to pyrethroids in Teso District**

			**% Mortality (Sample size)**			
**District**	**Cluster**	**Insecticide**	***An. gambiae s.s.***	***An. arabiensis***	**χ**^**2**^	**Pvalue**
Teso	Akiriamasi	Permethrin	50(8)	95(20)	7.89	0.005
Teso	Akiriamasit	Permethrin	85.7(7)	89.7(39)	0.1	0.75
Teso	Kaliwa	Permethrin	51.4(37)	7.1(14)	8.32	0.004
Teso	Kokare	Deltamethrin	85.4(48)	95.4(65)	3.4	0.07
Teso	Kokare	Permethrin	34.6(26)	71(31)	7.53	0.006
Teso	Koteko	Permethrin	82.4(51)	90.5(21)	0.76	0.21
Teso	Odioi	Deltamethrin	47.4(19)	83.3(6)	1.38	0.24
Teso	Odioi	Permethrin	35.1(97)	5.9(17)	5.78	0.02
Teso	Rwatama	Permethrin	83.5(79)	50(16)	4.62	0.03
Teso	Kengatunyi	Deltamethrin	28(43)	100(5)	1.86	0.22
Teso	Kaliwa	Deltamethrin	50(2)	44(18)	0.02	0.88
Teso	Kolanya	Deltamethrin	100(2)	78(72)	0.57	0.45
Teso	Rwatama	Deltamethrin	33(3)	81(77)	3.84	0.05
Teso	Adanya	Permethrin	100(6)	36(14)	7.01	0.008
Teso	Apatit	Permethrin	100(17)	0(0)	-	-
Teso	Apokor	Permethrin	96(47)	100(1)	0.04	0.83
Teso	Kabanyo	Permethrin	100(17)	100(2)		
Teso	Katelepai	Permethrin	50(42)	0(1)	0.98	0.32
Teso	Kengatunyi	Permethrin	86(79)	100(4)	0.64	0.42

## Discussion and conclusions

This study identified species specific phenotypic resistance of *An. gambiae* and *An. arabiensis* populations from 65 out of 80 clusters from 4 Districts in western Kenya. The four Districts in which this study was conducted are currently sites of widespread vector control using insecticide-based tools, ITNs and IRS. Resistance was widespread and heterogeneous with mosquito populations from 19 of 20 clusters in Bondo, 2 of 7 clusters in Nyando, 11 of 13 clusters in Rachuonyo and 14 of 15 clusters in Teso showing resistance to permethrin while 16 of 18 clusters in Bondo, 10 of 14 clusters in Rachuonyo, 5 of 18 clusters in Nyando and 6 of 6 clusters in Teso had resistance to deltamethrin. There was no correlation in cluster specific mortality of *An. arabiensis* between permethin and deltamethrin (*Z* = 2.9505 , *P* = 0.2483) indicating a difference in insecticidal efficacy between trans and alpha-cyano pyrethroids. For the small number of clusters in Teso where sufficient *An. gambiae* and *An. arabiensis* were obtained it was possible to determine 7 of the 17 tests that reached a conventional significance threshold (p < 0.05). 4 tests showed higher resistance in *An. arabiensis* and three in *An. gambiae*. Suggesting no consistent marked difference in resistance between species, which was confirmed by the signed ranked test.

Malaria vector control has heavily relied on the use of pyrethroids due to their efficacy and relatively low toxicity to non-target organisms [38,39]. The restricted and rather narrow range of insecticides for use in vector control necessitates strict monitoring and management of insecticide resistance within the control program to ensure sustenance of control [40-42]. Resistance to pyrethroids has emerged and is spreading at an alarming rate [15,43,44]. In this light, the Global Plan for Insecticide Resistance Management (GPIRM) document released by WHO in 2012 impresses upon the need to setup insecticide resistance monitoring programs where none exists and to scale-up monitoring efforts in areas that have implemented monitoring programs [42,45,46]. In Kenya, several studies have already documented insecticide resistance in multiple sites [5,23,24,47].

Nyando and Rachuonyo had, at the time of sample collection, yearly IRS programs in addition to the distribution of ITNs. Despite sustained vector control efforts employing pyrethroids since the early 2000s, vectors in Nyando demonstrated widespread susceptibility with only 5 of the 18 clusters tested for deltamethrin and 2 of the 7 clusters tested for permethrin having <90% mortality (WHO threshold for resistance) to the pyrethroids. The observation of low resistance despite long term insecticide use for public health had been made in previous studies in Asembo in Western Kenya and more previously in several sites in Tanzania [48,49]. Curiously, vectors in Teso and Bondo Districts, where only ITNs are the main malaria intervention, had the highest levels of insecticide resistance, suggesting that additional sources, may be contributing to the selection pressure for insecticide resistance. A study in Eastern Cote d'Ivoire actually observed pyrethroid resistance prior to the implementation of ITNs [50].

Whereas most resistance studies present susceptibility results representative of large administrative areas, usually Districts, this study presents cluster specific susceptibility data. Data from previous studies has shown sharply contrasting results between smaller sites within the administrative Districts. A study of *An. culicifacies* susceptibility to DDT in Baluchestan in Iran in 1972, revealed mortality between 16.4 and 42% in 5 villages within the same District [51]. In yet another study in two villages in Burkina Faso, Valle de Kou 5 and Valle de Kou 7 reported different mortalities of 73 and 100% to permethrin respectively [18]. In contrast, a study conducted in two villages in Apac District in Uganda, showed consistently similar mortality to permethrin over 3 sampling time points in ADA and ADB villages (99 and 98% mortality in January 2005, 93 and 92% mortality in September 2006 for *An. funestus* and 81 and 80% mortality in September 2006 in *An. gambiae* s.s) [52]. The range of mortality observed within and between Districts in this study is thus not without precedent. What remains to be seen is whether there is temporal stability in the estimates of mortality, which may allow us to determine the main drivers of the heterogeneity.

The distribution of the *An. gambiae* species in Teso further depicts the need for cluster-specific insecticide resistance monitoring. Clusters within the same District have varying distributions of *An. arabiensis* and *An. gambiae* and consequently varying insecticide resistance status. Given the differences in resting and feeding behaviour of the two sibling species, different transmission dynamics are expected to be observed in these clusters [53,54]. *An. arabiensis* has different vectorial behaviour compared to *An. gambiae* s.s., which is known to have stable behaviour and is more endophagic and endophilic, while An arabiensis may feed both indoors and outdoors but seems to be more exophagic and exophilic in the presence of pyrethroid resistance. The dominance reversal observed in western Kenya happened over many years, and since resistance is a fairly recent phenomenon, with time the impact of resistance on vector population structure may be observed.

Previous resistance work in Western Kenya has shown higher resistance in sites west, closer to the border with neighbouring Uganda where high resistance to pyrethroids has previously been detected [13,23,24,52]. Gene flow of resistance genes into bordering Districts may thus be a reason for the observed resistance phenotypes but this needs further genetic studies. Resistance has previously been linked to insecticide use in agriculture in several parts of the continent; Uganda [52,55], Burkina Faso [18] and other sites in sub-Saharan Africa [56-58]. In addition, resistance may be attributed to an increase in possession of ITNs and the implementation of IRS programs within the study Districts. For example, permethrin impregnated ITNs have been previously linked to a reduced susceptibility in *A. gambiae* s.s. though this elevation declined over time [5,49].

This baseline study provides a background for the study of insecticide resistance mechanisms in mosquito populations in the different clusters to enable effective management of insecticide resistance and at the same time facilitate continued vector control efforts [48,59]. Our study results show that insecticide resistance to pyrethroids is emerging within four Districts in Western Kenya. Apart from a call for more site-specific insecticide resistance monitoring, this study also emphasizes the need for further investigation into factors that can influence selection pressure in insecticide resistance in malaria vectors. Furthermore, monitoring on insecticide and pesticide use for agriculture should also be enhanced and a shift made for use of non-pyrethroid insecticides for IRS to preserve the pyrethroid class of insecticides for ITNs.

## Competing interests

The authors declare that they have no competing interests.

## Authors’ contributions

NMB, LK, FA, JV, MO, KN, DS, EM, LM, TK, MJD, IK and CM designed and developed the study. EO, NMB, LK, FA, JV, CO, KS, MJD and CM contributed to development of the protocol and data analysis. EO and KS performed the laboratory analysis of the samples. All authors took part in manuscript preparation, read and approved the final manuscript.

## Supplementary Material

Additional file 1 **Susceptibility status of mosquito populations to deltamethrin in the study clusters.** This data was used to populate Figure S1. Click here for file

Additional file 2 **Susceptibility status of mosquito populations to permethrin in the study clusters.** This data was used to populate Figure S2. Click here for file
