# Supplementary material for: Pyrethroid susceptibility of malaria vectors in four Districts of western Kenya
Source: Parasit Vectors. 2014 Jul 4;7:310. doi: 10.1186/1756-3305-7-310 (PMC4094666; doi:10.1186/1756-3305-7-310)
Supplement: Additional file 1 — Susceptibility status of mosquito populations to deltamethrin in the study clusters. This data was used to populate Figure S1. [file 1756-3305-7-310-S1.docx]

Additional File 1

| **District** | **Cluster** | **Intervention** | **Species** | **Total No. of Mosquitoes Tested** | **Alive** | **Dead** | **Proportion Dead** | **95% CI of proportion dead** |
| --- | --- | --- | --- | --- | --- | --- | --- | --- |
| Bondo | Abom | Deltamethrin | *An. arabiensis* | 13 | 6 | 7 | 0.54 | **0.29-0.77** |
| Bondo | Bar Kanyango | Deltamethrin | *An. arabiensis* | 28 | 12 | 16 | 0.57 | **0.39-0.73** |
| Bondo | Barchando | Deltamethrin | *An. arabiensis* | 17 | 7 | 10 | 0.59 | **0.36-0.78** |
| Bondo | Got Agulu | Deltamethrin | *An. arabiensis* | 10 | 4 | 6 | 0.6 | **0.31-0.83** |
| Bondo | Kagwa | Deltamethrin | *An. arabiensis* | 91 | 19 | 72 | 0.79 | **0.70-0.86** |
| Bondo | Kapiyo | Deltamethrin | *An. arabiensis* | 90 | 39 | 51 | 0.57 | **0.46-0.66** |
| Bondo | Kokwiri | Deltamethrin | *An. arabiensis* | 54 | 16 | 38 | 0.7 | **0.57-0.81** |
| Bondo | Mahaya | Deltamethrin | *An. arabiensis* | 77 | 25 | 52 | 0.68 | **0.56-0.77** |
| Bondo | Memba | Deltamethrin | *An. arabiensis* | 37 | 2 | 35 | 0.95 | **0.82-0.99** |
| Bondo | Naya | Deltamethrin | *An. arabiensis* | 7 | 4 | 3 | 0.43 | **0.16-0.75** |
| Bondo | Ndigwa | Deltamethrin | *An. arabiensis* | 82 | 9 | 73 | 0.89 | **0.80-0.94** |
| Bondo | Nyabera(Bondo) | Deltamethrin | *An. arabiensis* | 47 | 5 | 42 | 0.89 | **0.77-0.96** |
| Bondo | Nyangoma | Deltamethrin | *An. arabiensis* | 42 | 3 | 39 | 0.93 | **0.81-0.98** |
| Bondo | Omia Mwalo | Deltamethrin | *An. arabiensis* | 141 | 25 | 116 | 0.82 | **0.75-0.88** |
| Bondo | Ramba South | Deltamethrin | *An. arabiensis* | 46 | 5 | 41 | 0.89 | **0.77-0.95** |
| Bondo | Usigu | Deltamethrin | *An. arabiensis* | 124 | 19 | 105 | 0.85 | **0.77-0.90** |
| Bondo | Utonga | Deltamethrin | *An. arabiensis* | 114 | 31 | 83 | 0.73 | **0.64-0.80** |
| Bondo | Uyawi | Deltamethrin | *An. arabiensis* | 54 | 10 | 44 | 0.81 | **0.69-0.90** |
| Nyando | Tonde | Deltamethrin | *An. funestus* | 1 | 0 | 1 | 1 | **0.21-1.0** |
| Nyando | Tonde | Deltamethrin | *An. arabiensis* | 55 | 4 | 51 | 0.93 | **0.83-0.97** |
| Nyando | Ochoria | Deltamethrin | *An. arabiensis* | 38 | 5 | 33 | 0.87 | **0.73-0.94** |
| Nyando | Muhoroni East | Deltamethrin | *An. arabiensis* | 39 | 2 | 37 | 0.95 | **0.83-0.99** |
| Nyando | Koru | Deltamethrin | *An. arabiensis* | 36 | 0 | 36 | 1 | **0.90-1.0** |
| Nyando | Koru | Deltamethrin | *An. funestus* | 3 | 1 | 2 | 0.67 | **0.21-0.94** |
| Nyando | Kochogo North | Deltamethrin | *An. arabiensis* | 11 | 0 | 11 | 1 | **0.74-1.0** |
| Nyando | Kamswa North | Deltamethrin | *An. arabiensis* | 37 | 4 | 33 | 0.9 | **0.75-0.96** |
| Nyando | Kakola-Ombaka | Deltamethrin | *An. arabiensis* | 116 | 3 | 113 | 0.97 | **0.93-0.99** |
| Nyando | Kabar Central | Deltamethrin | *An. arabiensis* | 43 | 2 | 41 | 0.96 | **0.85-0.99** |
| Nyando | Kabar Central | Deltamethrin | *An. funestus* | 15 | 6 | 9 | 0.63 | **0.36-0.80** |
| Nyando | Jimo West | Deltamethrin | *An. arabiensis* | 50 | 0 | 50 | 1 | **0.93-1.0** |
| Nyando | Jimo West | Deltamethrin | *An. funestus* | 35 | 3 | 32 | 0.92 | **0.78-0.97** |
| Nyando | East Songhor | Deltamethrin | *An. arabiensis* | 17 | 2 | 15 | 0.88 | **0.66-0.97** |
| Nyando | Chemelil | Deltamethrin | *An. arabiensis* | 11 | 0 | 11 | 1 | **0.74-1.0** |
| Nyando | Border I | Deltamethrin | *An. arabiensis* | 48 | 1 | 47 | 0.98 | **0.89-1.0** |
| Nyando | Ayucha | Deltamethrin | *An. arabiensis* | 5 | 1 | 4 | 0.8 | **0.38-0.96** |
| Nyando | Agoro East | Deltamethrin | *An. arabiensis* | 29 | 1 | 28 | 0.97 | **0.83-0.99** |
| Rachuonyo | Kakwajuok Upper | Deltamethrin | *An. arabiensis* | 110 | 21 | 89 | 0.81 | **0.73-0.87** |
| Rachuonyo | Kalwal | Deltamethrin | *An. arabiensis* | 16 | 3 | 13 | 0.81 | **0.57-0.93** |
| Rachuonyo | Kamenya Central | Deltamethrin | *An. arabiensis* | 58 | 14 | 44 | 0.76 | **0.63-0.85** |
| Rachuonyo | Kamenya North | Deltamethrin | *An. arabiensis* | 19 | 2 | 17 | 0.89 | **0.69-0.97** |
| Rachuonyo | Kamser Seka | Deltamethrin | *An. arabiensis* | 41 | 13 | 28 | 0.68 | **0.53-0.80** |
| Rachuonyo | Kamwala | Deltamethrin | *An. arabiensis* | 38 | 7 | 31 | 0.82 | **0.67-0.91** |
| Rachuonyo | Kanyango I & II | Deltamethrin | *An. arabiensis* | 23 | 1 | 22 | 0.96 | **0.79-0.99** |
| Rachuonyo | Kawadhgone | Deltamethrin | *An. arabiensis* | 35 | 3 | 32 | 0.91 | **0.78-0.97** |
| Rachuonyo | Kobuya East | Deltamethrin | *An. arabiensis* | 47 | 14 | 33 | 0.7 | **0.56-0.81** |
| Rachuonyo | Koguta Homa Lime | Deltamethrin | *An. arabiensis* | 62 | 2 | 60 | 0.97 | **0.89-0.99** |
| Rachuonyo | Kogweno Oriang W | Deltamethrin | *An. arabiensis* | 48 | 9 | 39 | 0.81 | **0.68-0.90** |
| Rachuonyo | Kojuang | Deltamethrin | *An. arabiensis* | 34 | 4 | 30 | 0.88 | **0.73-0.95** |
| Rachuonyo | Kowili I & II | Deltamethrin | *An. arabiensis* | 23 | 1 | 22 | 0.96 | **0.79-0.99** |
| Rachuonyo | Ouko Ondege | Deltamethrin | *An. arabiensis* | 64 | 18 | 46 | 0.72 | **0.60-0.81** |
| Teso | Kaliwa | Deltamethrin | *An. gambiae s.l.* | 16 | 6 | 10 | 0.63 | **0.39-0.82** |
| Teso | Kengatunyi | Deltamethrin | *An. gambiae s.l.* | 44 | 15 | 29 | 0.66 | **0.51-0.78** |
| Teso | Kokare | Deltamethrin | *An. gambiae s.l.* | 101 | 22 | 79 | 0.78 | **0.69-0.85** |
| Teso | Kolanya | Deltamethrin | *An. gambiae s.l.* | 81 | 23 | 58 | 0.72 | **0.61-0.80** |
| Teso | Odioyi | Deltamethrin | *An. gambiae s.l.* | 34 | 15 | 19 | 0.56 | **0.39-0.71** |
| Teso | Rwatama | Deltamethrin | *An. gambiae s.l.* | 118 | 35 | 83 | 0.7 | **0.62-0.78** |
