# Supplementary material for: Pyrethroid susceptibility of malaria vectors in four Districts of western Kenya
Source: Parasit Vectors. 2014 Jul 4;7:310. doi: 10.1186/1756-3305-7-310 (PMC4094666; doi:10.1186/1756-3305-7-310)
Supplement: Additional file 2 — Susceptibility status of mosquito populations to permethrin in the study clusters. This data was used to populate Figure S2. [file 1756-3305-7-310-S2.docx]

Additional file 2

| **District** | **Cluster** | **Intervention** | **Species** | **Total No. of Mosquitoes Tested** | **Alive** | **Dead** | **Proportion Dead** | **95% CI of proportion dead** |
| --- | --- | --- | --- | --- | --- | --- | --- | --- |
| Bondo | Abom | Permethrin | An. arabiensis | 33 | 16 | 17 | 0.52 | **0.35-0.68** |
| Bondo | Bar Kanyango | Permethrin | An. arabiensis | 34 | 24 | 10 | 0.29 | **0.17-0.46** |
| Bondo | Barchando | Permethrin | An. arabiensis | 22 | 14 | 8 | 0.36 | **0.20-0.57** |
| Bondo | Got Agulu | Permethrin | An. arabiensis | 66 | 29 | 37 | 0.56 | **0.44-0.67** |
| Bondo | Kagwa | Permethrin | An. arabiensis | 107 | 66 | 41 | 0.38 | **0.30-0.48** |
| Bondo | Kapiyo | Permethrin | An. arabiensis | 56 | 17 | 39 | 0.7 | **0.57-0.80** |
| Bondo | Kokwiri | Permethrin | An. arabiensis | 49 | 15 | 34 | 0.69 | **0.55-0.80** |
| Bondo | Lieta | Permethrin | An. arabiensis | 75 | 14 | 61 | 0.81 | **0.71-0.89** |
| Bondo | Mahaya | Permethrin | An. arabiensis | 68 | 18 | 50 | 0.74 | **0.62-0.83** |
| Bondo | Masala | Permethrin | An. arabiensis | 12 | 1 | 11 | 0.92 | **0.65-0.99** |
| Bondo | Memba | Permethrin | An. arabiensis | 37 | 10 | 27 | 0.73 | **0.57-0.85** |
| Bondo | Naya | Permethrin | An. arabiensis | 21 | 10 | 11 | 0.52 | **0.32-0.72** |
| Bondo | Ndigwa | Permethrin | An. arabiensis | 104 | 24 | 80 | 0.77 | **0.68-0.84** |
| Bondo | Nyabera(Bondo) | Permethrin | An. arabiensis | 36 | 11 | 25 | 0.69 | **0.53-0.82** |
| Bondo | Nyangoma | Permethrin | An. arabiensis | 81 | 23 | 58 | 0.72 | **0.61-0.80** |
| Bondo | Omia Mwalo | Permethrin | An. arabiensis | 152 | 43 | 109 | 0.72 | **0.64-0.78** |
| Bondo | Ramba South | Permethrin | An. arabiensis | 67 | 28 | 39 | 0.58 | **0.46-0.69** |
| Bondo | Usigu | Permethrin | An. arabiensis | 259 | 111 | 148 | 0.57 | **0.51-0.63** |
| Bondo | Utonga | Permethrin | An. arabiensis | 117 | 68 | 49 | 0.42 | **0.33-0.51** |
| Bondo | Uyawi | Permethrin | An. arabiensis | 43 | 19 | 24 | 0.56 | **0.41-0.70** |
| Nyando | Tonde | Permethrin | An. arabiensis | 39 | 2 | 37 | 0.95 | **0.83-0.99** |
| Nyando | Muhoroni East | Permethrin | An. arabiensis | 14 | 2 | 12 | 0.86 | **0.60-0.96** |
| Nyando | Koru | Permethrin | An. arabiensis | 22 | 1 | 21 | 0.95 | **0.78-0.99** |
| Nyando | Kakola-Ombaka | Permethrin | An. arabiensis | 100 | 0 | 100 | 1 | **0.96-1.0** |
| Nyando | Kabar Central | Permethrin | An. arabiensis | 49 | 0 | 49 | 1 | **0.93-1.0** |
| Nyando | Jimo West | Permethrin | An. arabiensis | 31 | 6 | 25 | 0.82 | **0.64-0.91** |
| Nyando | Border I | Permethrin | An. arabiensis | 25 | 1 | 24 | 0.96 | **0.80-0.99** |
| Rachuonyo | Kagwa Seka | Permethrin | An. arabiensis | 13 | 5 | 8 | 0.62 | **0.36-0.82** |
| Rachuonyo | Kakwajuok Upper | Permethrin | An. arabiensis | 105 | 32 | 74 | 0.7 | **0.61-0.78** |
| Rachuonyo | Kamenya Central | Permethrin | An. arabiensis | 25 | 5 | 20 | 0.8 | **0.61-0.91** |
| Rachuonyo | Kamenya North | Permethrin | An. arabiensis | 46 | 13 | 33 | 0.72 | **0.57-0.83** |
| Rachuonyo | Kamser Seka | Permethrin | An. arabiensis | 31 | 5 | 26 | 0.84 | **0.67-0.93** |
| Rachuonyo | Kamwala | Permethrin | An. arabiensis | 13 | 6 | 7 | 0.54 | **0.29-0.77** |
| Rachuonyo | Kawadhgone | Permethrin | An. arabiensis | 15 | 0 | 15 | 1 | **0.80-1.0** |
| Rachuonyo | Kobuya East | Permethrin | An. arabiensis | 16 | 6 | 10 | 0.63 | **0.39-0.82** |
| Rachuonyo | Kobuya West | Permethrin | An. arabiensis | 15 | 6 | 9 | 0.6 | **0.36-0.80** |
| Rachuonyo | Koguta Homa Lime | Permethrin | An. arabiensis | 74 | 6 | 68 | 0.92 | **0.83-0.96** |
| Rachuonyo | Kogweno Oriang | Permethrin | An. arabiensis | 49 | 16 | 33 | 0.67 | **0.53-0.79** |
| Rachuonyo | Kojuang | Permethrin | An. arabiensis | 13 | 2 | 11 | 0.85 | **0.58-0.96** |
| Rachuonyo | Kowili I & II | Permethrin | An. arabiensis | 16 | 3 | 13 | 0.81 | **0.57-0.93** |
| Teso | Adanya | Permethrin | An. gambiae s.l. | 21 | 15 | 6 | 0.29 | **0.14-0.50** |
| Teso | Akiriamasi | Permethrin | An. gambiae s.l. | 15 | 5 | 10 | 0.67 | **0.38-0.89** |
| Teso | Akiriamasit | Permethrin | An. gambiae s.l. | 89 | 17 | 72 | 0.81 | **0.72-0.88** |
| Teso | Aloete | Permethrin | An. gambiae s.l. | 25 | 0 | 25 | 1 | **0.87-1.0** |
| Teso | Apatit | Permethrin | An. gambiae s.l. | 20 | 3 | 17 | 0.85 | **0.64-0.95** |
| Teso | Apokor | Permethrin | An. gambiae s.l. | 54 | 15 | 39 | 0.72 | **0.59-0.82** |
| Teso | Kakapel | Permethrin | An. gambiae s.l. | 9 | 6 | 3 | 0.33 | **0.12-0.65** |
| Teso | Kaliwa | Permethrin | An. gambiae s.l. | 101 | 25 | 76 | 0.75 | **0.66-0.83** |
| Teso | Katelepai | Permethrin | An. gambiae s.l. | 94 | 49 | 45 | 0.48 | **0.38-0.58** |
| Teso | Kengatunyi | Permethrin | An. gambiae s.l. | 107 | 14 | 93 | 0.87 | **0.79-0.92** |
| Teso | Kokare | Permethrin | An. gambiae s.l. | 127 | 37 | 90 | 0.71 | **0.62-0.78** |
| Teso | Kolanya | Permethrin | An. gambiae s.l. | 97 | 30 | 67 | 0.69 | **0.59-0.77** |
| Teso | Koteko | Permethrin | An. gambiae s.l. | 98 | 24 | 74 | 0.76 | **0.66-0.83** |
| Teso | Odioyi | Permethrin | An. gambiae s.l. | 99 | 62 | 37 | 0.37 | **0.28-0.47** |
| Teso | Rwatama | Permethrin | An. gambiae s.l. | 103 | 14 | 89 | 0.86 | **0.78-0.92** |
